# Rigid Esophagoscopy for Foreign Body Extraction: Results and Complications in the Endoscopic Era

**DOI:** 10.7759/cureus.53040

**Published:** 2024-01-27

**Authors:** Pedro L Alexandre, João V Pinto, Liliana Costa, Pedro Marques, Carla P Moura

**Affiliations:** 1 Otolaryngology, University Hospital Center of São João, Porto, PRT; 2 Surgery and Physiology, Faculty of Medicine, University of Porto, Porto, PRT; 3 Research, Center for Health Technology and Services Research (CINTESIS) University of Porto, Porto, PRT; 4 Otolaryngology, Centro Hospitalar do Tâmega e Sousa, Penafiel, PRT; 5 Otolaryngology and Genetics, University Hospital Center of São João, Porto, PRT; 6 Otolaryngology, Institute for Research and Innovation in Health (IS3) University of Porto, Porto, PRT

**Keywords:** esophageal perforation, swallowed foreign body, foreign body retrieval, esophageal foreign body, rigid esophagoscopy

## Abstract

Introduction

Rigid esophagoscopy (RE) has long been a part of otolaryngology practice. In the past decades, the procedure was less commonly performed due to the advances and availability of flexible endoscopic techniques. This study aims to describe the outcomes of RE performed to treat foreign body ingestion and to evaluate risk factors associated with postoperative complications.

Methods

Patients who underwent RE to treat foreign body ingestion in an otolaryngology emergency department of a Portuguese tertiary university hospital, between 2010 and 2020, were included. A total of 162 cases were analyzed, and data was collected retrospectively.

Results

The most common foreign bodies were meat bone (31.5%, n = 47), food impaction (28.8%, n = 43), and fish bone (19.5%, n = 29). The proximal esophagus was by far the most frequent location (80%, n = 118). Esophageal perforation occurred in 8% (13 patients), and there was a 2.5% (n = 4) mortality rate. The odds ratio of an esophageal perforation if the foreign body was completely or partially located outside the proximal esophagus was 4.67 times that of a foreign body exclusively in the proximal esophagus (OR = 4.67 [95% CI: 1.39-15.72]; p = 0.016; Fisher's exact test).

Conclusion

RE remains an effective and important technique in the management of ingested foreign bodies, particularly if endoscopic removal is unsuccessful. Foreign body location outside the proximal esophagus was associated with esophageal perforation.

## Introduction

Foreign body ingestion is a common cause of emergency visits and carries life-threatening risks mainly due to esophageal perforation [1,2]. Rigid esophagoscopy (RE) plays an important role in the management of foreign bodies, and the use of rigid instrumentation to observe and manipulate the esophageal lumen has been reported since the early nineteenth century, with Adolf Kussmaul (1822-1902) being credited with the first esophagoscopy [3].

RE remains part of otolaryngology practice until the present day as both the technique and instruments have been refined, allowing its application for diagnostic purposes (head and neck cancer staging) or as a therapeutic tool, for upper gastrointestinal (GI) tract (pyriform sinuses, retrocricoid region, and esophagus) foreign body extraction. Flexible endoscopic techniques have evolved, which provide many advantages such as requiring sedation only, allowing a magnified visualization of the esophagus, and causing less post-procedure discomfort [4]. On the other hand, RE allows more space for the passage of instruments, and the possibility of retrieving the foreign body within the esophagoscope protects the surrounding mucosa. If the otolaryngology examination does not detect any suspected foreign body in the oral cavity or the pharynx, it is usual, in many hospitals, to first perform a flexible endoscopy before a RE. The increased use of flexible endoscopic techniques led to a decrease in the number of REs performed with a noticeable impact on resident training [5]. While it is consensual that both techniques are equally effective, some series report higher perforation rates with RE. At the same time, a meta-analysis showed that both techniques appear to present similar complication rates, including perforation [2,6,7]. Hence, RE remains a useful and valuable choice in many situations, particularly after upper GI endoscopy failure to retrieve foreign bodies in the upper GI tract.

The present study aims to characterize a cohort of patients submitted to RE to treat foreign body ingestion and to study the risk factors associated with complications.

This article was previously presented as an oral communication at the 70th National Congress of the Portuguese Society of Otorhinolaryngology and Head and Neck Surgery, which was held from May 12 to May 14, 2023, in Algarve, Portugal.

## Materials and methods

Study design

A retrospective review of patients with suspected foreign body ingestion submitted to RE for foreign body extraction was conducted. Patients were observed at the otolaryngology emergency department of a Portuguese tertiary university hospital between January 2010 and December 2020. The present study was approved by the Ethics Committee of the University Hospital Center of São João (approval number 23/22).

Procedure and clinical data

RE was conducted under general anesthesia and orotracheal intubation in the emergency operating room (OR) by two otolaryngology surgeons (one senior and one resident, or two seniors) using a rigid esophagoscope with fiberoptic cable illumination (Roberts-Jesberg esophagoscopes, Karl Storz, Germany, Tuttlingen). Data collected included population demographics (age, sex, and relevant clinical history), patient's symptoms and clinical exam, type and location of foreign body, previous endoscopic removal attempt, and presurgical/iatrogenic complications. Complications were classified as minor (mucosal laceration, hematoma, bleeding, or dental avulsion) or major (esophageal perforation, abscess, pneumothorax, pneumomediastinum, mediastinitis, or death).

Statistical analysis

Statistical analysis was performed with SPSS Statistics for MacOS, version 27 (IBM Corp., Armonk, NY). Normality was evaluated with the Kolmogorov-Smirnov test or skewness/kurtosis, and statistical associations were evaluated with Fisher's exact test/chi-square test as appropriate. Statistical significance was considered if the p-value was less than 0.05.

## Results

Population

A total of 162 patients were identified, among which 82 were male and 80 were female. The mean age was 60.2 ± 17.5 years. Almost half of the patients, 42% (n = 68), were admitted to the emergency department during the first 24 hours after foreign body ingestion, the longest delay being seven days. Clinical symptoms were foreign body sensation in the throat (63.2%, n = 86), dysphagia (33.8%, n = 46), odynophagia (32.4%, n = 44), and thoracic discomfort/pain (7.4%, n = 12). In 31.4% of patients (n = 44), sialorrhea or hypopharyngeal salivary stasis was observed on nasal fibroscopy. An upper GI endoscopy was performed by the gastroenterology team, before the RE, in 132 cases (81.5%), without success in retrieving the foreign body. There were two deliberate ingestions of foreign material, with both patients presenting neuropsychiatric conditions. Population demographics and clinical data are shown in Table 1.

**Table 1:** Population demographics and clinical data RE: Rigid esophagoscopy.

Population	162
Age in years, mean (SD)	60.2 (17.5)
Male, n (%)	82 (50.6%)
Female, n (%)	80 (49.4%)
Time from ingestion until medical observation, n (%)	
<24 hours	68 (42%)
>72 hours	18 (11%)
Clinical presentation, n (%)	
Foreign body sensation	86 (63.2%)
Dysphagia	46 (33.8%)
Odynophagia	44 (32.4%)
Sialorrhea or salivary stasis on nasal fibroscopy	44 (31.4%)
Thoracic discomfort/pain	12 (7.4%)
Fever	5 (3.1%)
Relevant comorbidities, n (%)	
Psychiatric disorders	18 (11.0%)
Neurologic disorders	16 (9.9%)
Diabetes mellitus type 2	13 (8.1%)
Gastrointestinal disorders	12 (7.4%)
Upper GI endoscopy attempt prior to RE, n (%)	
Unsuccessful	132 (81.5%)
Not performed	26 (16%)
Missing data	4 (2.5%)
Foreign body on RE, n (%)	
Foreign body identified	149 (92%)
No foreign body identified	13 (8.0%)

Foreign body characteristics

**Figure 1:**
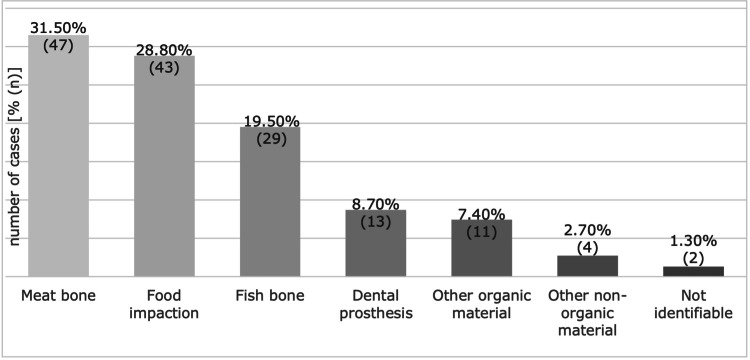
Types of foreign bodies The image shows the foreign bodies identified during rigid esophagoscopy in the order of frequency.

**Figure 2:**
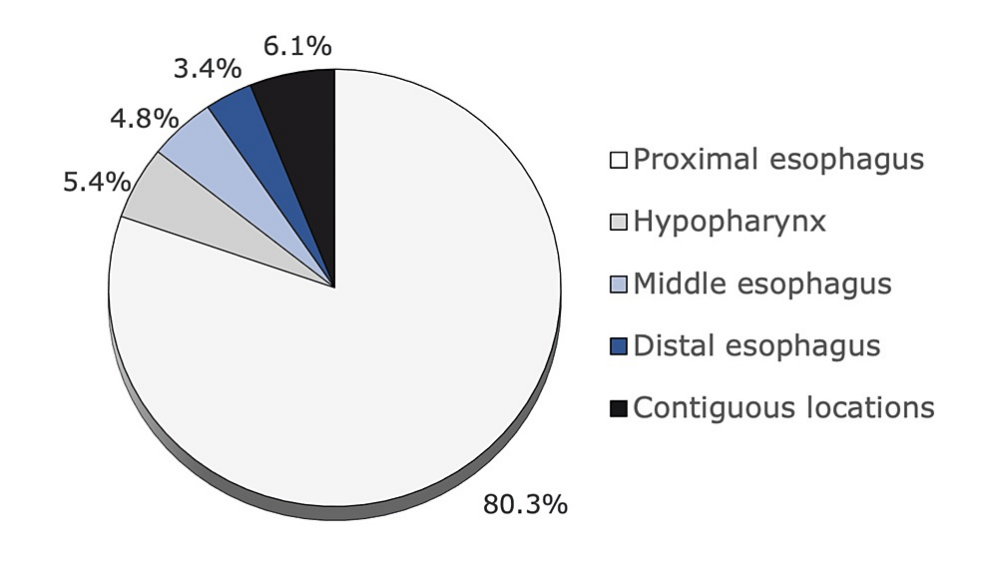
Foreign body location The proximal esophagus was the most common location (80,3%, n = 118), followed by contiguous locations (6,1%, n = 9), hypopharynx (5,4%, n = 8), middle esophagus (4.8%, n = 7), and distal esophagus (3.4%, n = 5).

Of the 162 REs performed, a foreign body was identified in 149 (92%) cases. The most common was meat bone (31.5%, n = 47), followed by food impaction (28.8%, n = 43) and fish bone (19.5%, n = 29) (Figure 1). Foreign bodies classified as other organic material include a bivalve shell, tooth, and cork, while non-organic include a metal sink plug and polyurethane foam. The most common location was, by far, the proximal esophagus (80%, n = 118). Food impaction in more than one segment of the esophagus (middle and distal, proximal and middle, or the whole esophagus), or a foreign body extending from the hypopharynx to the proximal esophagus (most commonly a fish bone), were classified as in contiguous locations (Figure 2).

Rigid esophagoscopy results and complications

Of these 149 patients, RE was successful in extracting the foreign bodies in 144 patients (96.6%). The remaining five cases underwent an upper GI flexible endoscopy under general anesthesia (three patients) or abdominal laparotomy (two patients). Of these five cases, three had a foreign body in the middle/distal esophagus, one had an esophageal stenosis not passable with the esophagoscope, and one had a dental prosthesis lodged in the proximal esophagus. In the total cohort (n = 162), esophageal mucosa laceration was the most common minor complication (38.9%, n = 63). Fifteen patients experienced major complications. All of them, except for two (a mediastinitis and a septic shock), were associated with an esophageal perforation (Table 2). The median length of stay was 2.5 days. Nineteen patients (11.7%) required postoperative vigilance and/or critical care in an intensive care unit. Four patients (2.5%) required a second procedure to treat complications (Table 3). The overall mortality rate of the cohort was 2.5% (four patients).

**Table 2:** Preoperative or iatrogenic complications This table shows the individual frequency of each complication (number and percentage).

Minor complications	n (%)
Mucosal laceration	63 (38.9%)
Endoluminal bleeding/hematoma	17 (9.3%)
Mucosal edema	6 (3.7%)
Dental avulsion	3 (1.9%)
Major complications	n (%)
Esophageal perforation	13 (8%)
Abscess	5 (3.1%)
Death	4 (2.5%)
Pneumothorax/hydropneumothorax	3 (1.9%)
Pneumomediastinum	3 (1.9%)
Cervical emphysema	2 (1.2%)
Mediastinitis	2 (1.2%)
Septic shock	1 (0.6%)

**Table 3:** Reinterventions A summary of the four cases that required a second intervention to treat major complications is given in this table.

Foreign body	Location	Complications	Procedure
Bone	Proximal esophagus	Esophageal perforation + retropharyngeal abscess	Multiple cervicotomies with parapharyngeal abscess drainage
Food impaction	Proximal esophagus	Esophageal perforation + paraesophageal abscess + pneumomediastinum	Transhiatal drainage of a paraesophageal abscess and endoscopic esophageal stent placement
Food impaction	Middle and distal esophagus	Esophageal perforation + hydropneumothorax + mediastinitis	Esophageal exclusion and jejunostomy, distal esophagectomy, esophagostomy closure
Food impaction	Proximal, middle, and distal esophagus	Esophageal perforation + hydropneumothorax	Endoscopic esophageal perforation repair and stent placement

All cases of esophageal perforation, except one, occurred in REs in which a foreign body was identified. Of the 13 patients with no foreign body identified during the RE, 11 had been previously submitted to an upper GI, which described the presence of a foreign body (but extraction was not successful/attempted). Statistical analysis showed that the odd of esophageal perforation if the foreign body was completely or partially located outside the proximal esophagus was 4.67 times that of a foreign body exclusively in the proximal esophagus (OR = 4.67 [95% CI: 1.39-15.72], p = 0.016, Fisher's exact test). The group with esophageal mucosa laceration had a higher complication rate (14.3%) than the group with no laceration (6.2%), but no meaningful difference could be proven (p = 0.085, chi-square test). The meantime, in days, between foreign body ingestion and treatment was higher in patients who developed major complications versus those who did not (1.63 vs 0.91 days), but sample size limited further statistical analysis.

## Discussion

After the advent of flexible upper digestive endoscopy and with the availability of more vast and advanced endoscopic techniques, suspected esophageal foreign bodies are usually referred to an upper GI endoscopy. In this study’s institution, after observation by the otolaryngology team and whenever feasible, this is likely the usual procedure. In 81.5% of the patients, foreign body extraction was not possible with the flexible endoscopic procedure. This means that the majority of the RE was performed in a previously manipulated esophagus, which can contribute to the complications found, both minor and major. Esophageal perforation rates after RE vary in the published literature. Hsu et al. had a 0.2% perforation rate, Loh et al. reported esophageal perforation in 6.2%, and Athanassiadi et al. found only one case (0.3%) [8-10].In this series, the rate of esophageal perforation was 8%, which sets it at a higher limit than those reported in the literature. It is noteworthy to mention that of the 13 patients with no foreign body identified in the RE, 11 had been previously submitted to an upper GI that described its presence. The most conceivable explanation is that a distal progression of the foreign body to the gastric chamber occurred in the period between both procedures.

The most common foreign body (bone) and its location (proximal esophagus) are comparable to those described in previous studies, although some series report fish bone as the most common foreign body [10,11]. The RE success rate was high (96.6%), with only five cases requiring conversion to an alternative technique. In three of these cases, upper GI endoscopy was performed in the OR with general anesthesia. Two of them had already been submitted to a previous upper GI endoscopy (without general anesthesia). In the other two cases, general surgery performed an abdominal laparotomy (1.3% laparotomy necessity rate). The first case was a dental prosthesis lodged in the proximal esophagus, which was impossible to extract superiorly, so it progressed to the distal end of the esophagus. An open gastrostomy was performed, and the prosthesis mobilized to the stomach and was removed. The patient evolved with no complications. The second refers to a case of food impaction in the distal esophagus. An open gastrotomy was performed to treat the impaction, followed by bipolar exclusion of the esophagus (and feeding jejunostomy) and esophageal perforation repair via thoracotomy. The patient evolved poorly and died due to a postoperative infection. All cases of esophageal perforation, except for one, occurred in RE in which a foreign body was identified and extracted.

In the last few years, the increased availability and advances of flexible endoscopic techniques have led to a decrease in the number of rigid esophagoscopies performed. This gradual change in the clinical practice has implications for the skills of otolaryngology residents, who still need to be comfortable with a technique that carries potentially serious risks even when performed at the hands of an experienced surgeon. In the present cohort, RE was confirmed to be an effective and safe method to extract an ingested foreign body, but the perforation and mortality rates (8% and 2.5%, respectively) should be kept in mind while managing these patients. The retrospective nature of this study may have limited data collection, which is the main limitation. A large-scale study, ideally multicenter, with a higher absolute number of patients with major complications could also give further conclusions.

## Conclusions

RE remains an important tool in the management of patients with an esophageal foreign body. There was an association between esophageal perforation and foreign body location outside the proximal esophagus. The advances in upper GI endoscopy techniques and their availability signify a future trend that reserves RE to the most challenging cases, which could be associated with a significant rate of complications.
